# Cardioprotective effects of 5‐hydroxymethylfurfural mediated by inhibition of L‐type Ca^2+^ currents

**DOI:** 10.1111/bph.13967

**Published:** 2017-09-09

**Authors:** G Wölkart, A Schrammel, C N Koyani, S Scherübel, K Zorn‐Pauly, E Malle, B Pelzmann, M Andrä, A Ortner, B Mayer

**Affiliations:** ^1^ Institute of Pharmaceutical Sciences, Department of Pharmacology and Toxicology University of Graz Graz Austria; ^2^ Institute of Molecular Biology and Biochemistry Medical University of Graz Graz Austria; ^3^ Institute of Biophysics Medical University of Graz Graz Austria; ^4^ Department of Thoracic and Cardiovascular Surgery Klinikum Klagenfurt Klagenfurt Austria; ^5^ Institute of Pharmaceutical Sciences, Department of Pharmaceutical Chemistry University of Graz Graz Austria

## Abstract

**Background and Purpose:**

The antioxidant 5‐hydroxymethylfurfural (5‐HMF) exerts documented beneficial effects in several experimental pathologies and is currently tested as an antisickling drug in clinical trials. In the present study, we examined the cardiovascular effects of 5‐HMF and elucidated the mode of action of the drug.

**Experimental Approach:**

The cardiovascular effects of 5‐HMF were studied with pre‐contracted porcine coronary arteries and rat isolated normoxic‐perfused hearts. Isolated hearts subjected to ischaemia/reperfusion (I/R) injury were used to test for potential cardioprotective effects of the drug. The effects of 5‐HMF on action potential and L‐type Ca^2+^ current (I_Ca,L_) were studied by patch‐clamping guinea pig isolated ventricular cardiomyocytes.

**Key Results:**

5‐HMF relaxed coronary arteries in a concentration‐dependent manner and exerted negative inotropic, lusitropic and chronotropic effects in rat isolated perfused hearts. On the other hand, 5‐HMF improved recovery of inotropic and lusitropic parameters in isolated hearts subjected to I/R. Patch clamp experiments revealed that 5‐HMF inhibits L‐type Ca^2+^ channels. Reduced I_Ca,L_ density, shift of I_Ca,L_ steady‐state inactivation curves toward negative membrane potentials and slower recovery of I_Ca,L_ from inactivation in response to 5‐HMF accounted for the observed cardiovascular effects.

**Conclusions and Implications:**

Our data revealed a cardioprotective effect of 5‐HMF in I/R that is mediated by inhibition of L‐type Ca^2+^ channels. Thus, 5‐HMF is suggested as a beneficial additive to cardioplegic solutions, but adverse effects and contraindications of Ca^2+^ channel blockers have to be considered in therapeutic application of the drug.

**Abbreviations**
+dP/dt_max_: maximum rate of left‐ventricular pressure increase
5‐HMF: 5‐hydroxymethylfurfural
AP: action potential
APD: action potential duration
*C*_m_: cell membrane capacitance
CPP: coronary perfusion pressure
−dP/dt_max_: maximum rate of left‐ventricular pressure decrease
GPV: guinea pig ventricular
HbS: sickle haemoglobin
I/R: ischaemia/reperfusion
I_Ca,L_: L‐type Ca^2+^ current
I_K_: potassium current
I_ss_: steady‐state current
LVDevP: left‐ventricular developed pressure
LVEDP: left‐ventricular end‐diastolic pressure

## Introduction

The furan derivative 5‐hydroxymethylfurfural (5‐HMF) is present in a wide variety of foods and beverages including milk, alcoholic drinks, fruit juice, honey and bakery products. Formation of 5‐HMF occurs in the course of thermal processing, non‐enzymic browning reactions or prolonged storage of food. The compound is produced from reducing sugars (fructose, saccharose and to a lesser degree glucose) either *via* the Maillard reaction in the presence of proteins (Antal *et al.,* 1990) or by direct acid‐catalysed thermal dehydration of monosaccharides. 5‐HMF was also identified in chewing tobacco and cigarette smoke (Black, 1966; Baldwin *et al.,* 1994). Average daily intake has been estimated to vary between 30 to 60 mg (0.5 ± 1 mg·kg^−1^ body weight), but consumption of dried fruits, caramel products and beverages made from plums may lead to intake of up to 350 mg of 5‐HMF per day (Abraham *et al.,* 2011). *In vitro* studies revealed a moderate genotoxic and mutagenic potential of 5‐HMF at very high concentrations of the compound (Janzowski *et al.,* 2000). In a more recent study from Abraham *et al*. (2011), no genotoxic effects were observed *in vivo*. Based on these results, the authors excluded any health risk of 5‐HMF at average food intake. However, concentration limits for the compound have been introduced for parenteral glucose preparations in several pharmacopoeias.

Administration of 5‐HMF in combination with α‐ketoglutaric acid as a dietary supplement was reported to improve aerobic exercise performance of athletes under hypoxia, an effect that was ascribed to the antioxidative capacity of the product (Gatterer *et al.,* 2013). Due to this antioxidative potential, a formulation of 5‐HMF and α‐ketoglutaric acid (Sanopal®, C.Y.L Pharmazeutika GmbH, Austria) has been recommended as dietary supplement for fast‐track surgical patients to reduce perioperative complications and to shorten rehabilitation (Matzi *et al.,* 2007).

From a therapeutic perspective, 5‐HMF has recently gained attention as potential antisickling drug (Abdulmalik *et al.,* 2005; Safo and Kato, 2014). Sickle cell‐diseased patients express abnormal (sickling) haemoglobin (HbS) that affects the morphological and rheological characteristics of red blood cells and may lead to occlusion of small vessels and capillaries under hypoxic conditions. Oral administration of 5‐HMF to transgenic sickle mice resulted in inhibition of erythrocyte sickling and prolongation of survival time under severe hypoxia. This beneficial effect was ascribed to selective binding of 5‐HMF to HbS, thereby altering the oxygen affinity of haemoglobin. Thus, 5‐HMF has already completed preclinical testing and entered clinical trials. Moreover, beneficial effects of 5‐HMF have been reported for conditions of acute hypobaric hypoxia (Mariacher *et al.,* 2014), alcoholic liver oxidative injury (Li *et al.,* 2015), cerebral ischaemia (Ya *et al.,* 2017) and cognitive impairments (Lee *et al.,* 2015).

Oxidative stress, that is, supraphysiological production of (primarily) superoxide anion, is considered to be a major trigger for the development and progression of cardiovascular pathologies such as vascular endothelial dysfunction, hypertension and cardiac hypertrophy. Moreover, metabolic disorders such as type 2 diabetes as well as the process of ageing have been frequently associated with increased superoxide production. The present study was initially designed to see how the antioxidative capacity of 5‐HMF affects cardiovascular function but surprisingly revealed cardioprotective effects that are apparently mediated by inhibition of voltage‐gated L‐type Ca^2+^ channels. Obviously, the observed low potency of 5‐HMF will not restrict use of the drug as an additive to cardioplegic solutions, but inhibition of Ca^2+^ channels may also come into effect *in vivo* upon application of commercial formulations containing high amounts of 5‐HMF.

## Methods

### Animals and tissue collection

All animal care and experimental procedures were in compliance with the Austrian law on experimentation with laboratory animals (last amendment, 2013) based on the European Union guidelines for the Care and the Use of Laboratory Animals (European Union Directive 2010/63/EU). Animal studies are reported in compliance with the ARRIVE guidelines (Kilkenny *et al.,* 2010; McGrath and Lilley, 2015).

Rats (Sprague–Dawley; total number: 18*)* and guinea pigs (Dunkin‐Hartley; total number: 16) of either sex were purchased from Charles River Laboratories (Sulzfeld, Germany) and housed at the animal facility at the Institute, until they were used for the study, at 12–16 weeks of age. Rats were kept in approved conventional polycarbonate cages (Ehret, Emmerdingen, Germany), two to three animals per cage with dust‐free laboratory bedding and enrichment (nesting material and rodent tunnels; from Abedd GmbH, Vienna, Austria). Guinea pigs were kept in big floor cages provided with litter and hiding boxes. They were fed standard chow diet [Altromin 1324 (rats) or Altromin 3023 (guinea pigs) from Altromin, Lage, Germany] and provided drinking water *ad libitum*. Animals were maintained at 23 ± 1°C, with a relative humidity of 50–70% and kept on a regular 12 h dark/light cycle.

Hearts of rats and guinea pigs were excised under deep anaesthesia induced by i.p. injection of ketamine/xylazine (80 mg·kg^−1^ ketamine, 10 mg·kg^−1^ xylazine mixture). Depth of anaesthesia was monitored by toe pinch reflex. Porcine hearts were obtained from a local abattoir. At the abattoir, pigs were rendered unconscious by electrical stunning carried out by trained stuff and thereafter exsanguinated by cutting the throat. Hearts were then removed and rapidly transported to the laboratory.

### Validity of animal species and model selection

Heart studies were performed with Sprague–Dawley rats, a species that has been widely used in the literature as source of cardiovascular tissue. However, to reduce animal suffering, detailed relaxation studies were performed with isolated coronary arteries from pigs, which are easily available from the abattoir, preventing unnecessary killing of laboratory animals. Because isolation of guinea pig ventricular (GPV) cells is well established in the laboratory and cells were readily available from other studies, guinea pig cardiomyocytes were used for electrophysiology experiments. As Ca_v_1.2 channels carry the L‐type Ca^2+^ current (I_Ca,L_) in guinea pig (Feng *et al.,* 2014; Nassal *et al.,* 2016) as well as rat ventricular myocytes (Schwoerer *et al.,* 2013; Liu *et al.,* 2014b), it is reasonable to assume that 5‐HMF affects this current in a similar way in both species. To exclude the possibility that effects observed in guinea pig cells were absent in rat ventricular myocytes, key experiments were repeated with rat isolated cardiomyocytes confirming the results with cells isolated from guinea pigs (not shown).

### Isometric tension vasomotor studies

The right coronary artery was carefully explanted from porcine hearts, cleaned from connective tissue and immediately used for assessment of vessel function. For isometric tension measurements, vessel rings were suspended in 5 mL organ baths containing oxygenated Krebs–Henseleit buffer (concentrations in mM: NaCl 118.4, NaHCO_3_ 25, KCl 4.7, KH_2_PO_4_ 1.2, CaCl_2_ 2.5, MgCl_2_ 1.2, D‐glucose 11; pH 7.4), as previously described in detail (Neubauer *et al.,* 2015). After equilibration for 90 min at the optimal resting tension of 20 mN, maximal contractile activity was determined with a depolarizing solution containing 60 mM K^+^. Rings that did not elicit adequate and stable contraction to high K^+^ were considered to be damaged and omitted from the study. After washout, tissues were precontracted with the thromboxane mimetic 9,11‐dideoxy‐9α,11α‐epoxy‐methanoprostaglandin F_2α_ (U‐46619; 50 nM) to an equivalent level (i.e. ~60% of maximal contraction obtained with high K^+^). When contraction had reached a stable plateau (~after 20 min), cumulative concentration–response curves were established with 5‐HMF. A potential role of the endothelium in the observed relaxation was examined in endothelium‐denuded rings. The endothelium was removed by gently rubbing the intimal surface with a wooden stick. The effectiveness of endothelial removal was confirmed by the lack of bradykinin‐induced relaxation. Where indicated, the effects of several antagonists on 5‐HMF‐induced relaxation were tested in paralleled rings that had been pre‐incubated with the corresponding inhibitors for at least 30 min. Effects to each agonist are expressed as percentages of relaxation of the U‐46619‐induced contraction.

### Langendorff perfusion of isolated hearts

The cardiac effects of 5‐ HMF were investigated in isolated hearts from adult Sprague–Dawley rats (250–350 g) of either sex. Heart perfusion experiments were performed as previously described (Wölkart *et al.,* 2007) with slight modifications. Immediately after removal, hearts were mounted on a perfusion apparatus (Hugo Sachs Elektronik/Harvard Instruments, March‐Hugstetten, Germany) and retrogradely perfused with Krebs–Henseleit bicarbonate buffer (pH 7.4, 37°C) at a constant flow of 10 mL·min^−1^·g^−1^ heart wet weight. A fluid‐filled balloon was inserted into the left ventricle and connected to a pressure transducer. The following cardiac parameters were monitored in unpaced hearts: left‐ventricular developed pressure (LVDevP), left‐ventricular end‐diastolic pressure (LVEDP; set at 5 mmHg at the beginning of the experiment), maximum rate of left‐ventricular pressure increase and decrease (+dP/dt_max_, −dP/dt_max_), coronary perfusion pressure (CPP; an index of coronary arterial function) and heart rate (electronically derived from the pressure signal).

In the ischaemia/reperfusion (I/R) experiments, ischaemic contracture was taken as the rise in diastolic tension after onset of ischaemia. It was calculated as the ratio of peak diastolic pressure reached during ischaemia over pre‐ischaemic LVDevP (100% value) and is expressed as percentage. Time‐to‐onset of ischaemic contracture was defined as the time when diastolic pressure reached 5% of control LVDevP. Time‐to‐resumption of sinus rhythm was assessed as the period after onset of reperfusion when identifiable systolic and diastolic pressures appeared that were not accompanied by the occurrence of massive arrhythmias.

Hearts were subjected to the following experimental protocols:

- Perfusion for 30 min to establish stable baseline conditions, followed by bolus addition of increasing concentrations of 5‐HMF (0.1–10 mM; non‐cumulative dosing) *via* a sideline to establish a concentration response curves.
- After equilibration, vehicle or 5‐HMF (5 mM) was added to the perfusion medium for 10 min. Thereafter, hearts were subjected to 20 min of no‐flow ischaemia at 36–37°C and reperfused with vehicle or 5‐HMF containing buffer for 30 min.

### Isolation of primary guinea pig ventricular (GPV) cardiomyocytes

GPV myocytes were isolated as described previously (Pelzmann *et al.,* 2003). Briefly, guinea pigs were killed, hearts were quickly removed, mounted on a Langendorff apparatus and the coronary system was perfused with buffer (composition in mM: NaCl 126, KCl 4.7, KH_2_PO_4_ 1.2, MgSO_4_ 2.5, NaHCO_3_ 2.49, HEPES/Na^+^ 0.5 and D(+)‐glucose 5.45, pH 7.4 adjusted with NaOH) containing 2 mM pyruvic acid, 1 mg·mL^−1^ bovine serum albumin and 100 IU·mL^−1^ collagenase (Worthington CLS 2) at 37°C (Scheruebel *et al.,* 2014). After enzymatic digestion, cardiomyocytes were isolated from ventricles and Ca^2+^ concentration was raised stepwise to 1.8 mM. Cells were transferred to cell culture medium (M199) containing 50 IU·mL^−1^ penicillin and 50 μg·mL^−1^ streptomycin and maintained at 37°C under 5% CO_2_. All experiments were performed on the day after isolation.

### Electrophysiological recordings and analysis

Electrophysiological recordings were performed in the whole‐cell configuration of the patch‐clamp technique using the amplifiers List L/M‐EPC 7 (List, Darmstadt, Germany) and Axopatch 200B (Molecular Devices, CA, USA) and the A/D – D/A converters Digidata 1322A and Digidata 1200 (Molecular Devices LLC). Patch pipettes with tip‐resistance of 2–3 MΩ were used. The pCLAMP software (Molecular Devices LLC) was used for data acquisition and analyses. Only quiescent rod‐shaped cardiomyocytes were used for electrophysiological experiments. Ion currents were normalized to cell membrane capacitance (*C*_m_) and expressed as pA/pF to compensate for cell size variations. *C*_m_ was determined by integration of the capacitive transient elicited by a 10 mV hyperpolarizing step from −50 mV. *C*_m_ and series resistance (*R*_s_, by at least 50%) were compensated, and leak subtraction was not performed. Current recordings were started 4 min after rupture of the cell membrane patch to allow equilibration of the pipette solution with the cytosol. Ion currents and action potentials (APs) were recorded at 36–37°C.

To record APs and steady‐state current (I_ss_), cardiomyocytes were superfused with the standard external solution containing (in mM) NaCl 137, KCl 5.4, CaCl_2_ 1.8, MgCl_2_ 1.1, NaHCO_3_ 2.2, NaH_2_PO_4_ 0.4, HEPES/Na^+^ 10, D(+)‐glucose 5.6, adjusted to a pH of 7.4 with NaOH. Pipettes were filled with an internal solution containing (in mM) KCl 110, ATP/K^+^ 4.3, MgCl_2_ 2, CaCl_2_ 1, EGTA 11, HEPES/K^+^ 10, adjusted to a pH of 7.4 with KOH (estimated free [Ca^2+^] < 10^−8^ M). For AP recordings, cells were stimulated with minimal suprathreshold current pulses (5 ms) at a frequency of 1 Hz, as previously described (Zorn‐Pauly *et al.,* 2005). pClamp data were imported into MATLAB® (R2016a), and the AP parameters of 10 consecutive APs were algorithmically analysed and averaged whereby the first 10 APs were excluded from analysis in order to exclude any initial transient behaviour. I_ss_ recordings were performed using voltage ramps from −100 to +60 mV (20 s duration). Each ramp current was discretized at 10 mV intervals by averaging three adjacent data points.

In order to study I_Ca,L_ without contamination of Na^+^ current (I_Na_), a 50 ms prepulse to −40 mV from a holding potential of −80 mV was used to inactivate I_Na_. Potassium currents (I_K_) were suppressed by substituting K^+^ with Cs^+^ in both, external and internal solution. I_Ca,L_ was elicited by a train of voltage pulses from −40 to +90 mV (10 mV interval, 400 ms), and the amplitude was calculated as the difference between the peak inward current and the current at the end of the pulse.

To calculate I_Ca,L_ conductance (g_Ca,L_), peak values of I_Ca,L_ were divided by the driving force. I_Ca,L_ reversal potential was not significantly different between control and treated cardiomyocytes and the mean value amounted +54 mV. For determination of steady‐state activation curves, conductances were normalized to the highest value and fitted according to Boltzmann function

(1) $$d_{\infty }=\frac{1}{\left\{1+\exp\left[\frac{V_{\frac{1}{2}\mathrm{act}}-V}{k}\right]\right\}}$$

where V is the membrane potential, V_1/2act_ is the membrane potential of half maximal activation and k is the slope of the activation curve.

For the determination of steady‐state inactivation, I_Ca,L_ was activated with test pulses to +10 mV (400 ms), which were preceded by conditioning prepulses from −45 to +50 mV (5 mV increment, 400 ms). The amplitude of I_Ca,L_ elicited by the test pulse was normalized to the highest value and plotted as a function of prepulse potential. Normalized values (−45 to +10 mV prepulse potential) were fitted according to Boltzmann function

(2) $$f_{\infty }=\frac{1}{\left\{1+\exp\left[\frac{V-V_{\frac{1}{2}\text{inact}}}{k}\right]\right\}}$$

where V is the membrane potential, V_1/2 inact_ is the membrane potential of half maximal inactivation and k is the slope of the inactivation curve.

The time dependence of I_Ca,L_ recovery from inactivation was examined using a double‐pulse protocol. Two depolarizing pulses to +10 mV with varying inter‐pulse intervals (10–5000 ms) were applied from a holding potential of −40 mV every 8 s. The extent of recovery at each inter‐pulse interval was analysed by expressing the amplitude of I_Ca,L_ in response to the test pulse as a fraction of the calcium current amplitude elicited by the conditioning pulse. Half‐recovery time was used to evaluate drug effects.

### Randomization and blinding

Porcine hearts were collected by workers at the abattoir, and the researcher had no influence on the selection of hearts. Laboratory animals were also randomly assigned to experiments. Experiments were not blinded in this study, as investigators were responsible for conducting and analysing experiments. Blinding is not a usual procedure for this form of study and cannot be applied retrospectively. To reduce experimental bias, data analysis was not performed until the complete data set had been collected, and no complete recording was excluded.

### Data and statistical analysis

Data and statistical analyses comply with the recommendations on experimental design and analysis in pharmacology (Curtis *et al*., 2015). Mechanical responses of coronary arteries were recorded as force (mN) and normalized to U‐46619‐induced contraction. Effects to each agonist were expressed as percentage of relaxation of U‐46619‐induced contraction. The 5‐HMF concentration–response curves were fitted to a Hill‐type model giving estimates of agonist potency (EC_50_) and efficacy (E_max_). Ion currents were normalized to *C*_m_ and expressed as pA/pF to compensate for cell size variations. Measurements on multiple cells or vessel rings derived from a single animal were averaged and counted as an individual experiment (*n* = 1). Group sizes of the different experiments are provided in the corresponding figure legend. In compliance with the laws on experimentation with laboratory animals, the minimum number of animals to achieve statistical significance has been used. All data are presented as mean values ± SEM of *n* experiments. Approximate normal distribution of data was determined by visual (histograms and normal Q‐Q plots), and numerical (z‐value of skewness and kurtosis; *P* value of Shapiro–Wilk test) investigation and homogeneity of variance was checked by Levene's test. Significance between means was assessed with the use of paired or unpaired Student's *t*‐test. Where appropriate, ANOVA with *post hoc* Bonferroni–Dunn test was used for comparison between groups. Data from APD and I_ss_ measurements showed a signficant time‐matched rundown effect and were therefore analysed using ANCOVA comparing the effect of 5‐HMF versus rundown by adjusting the matched control values (covariates). ANCOVA was only applied when covariates and regression slopes were not different between the compared groups. Changes in APD are shown as percentage change to make the 5‐HMF effect clearly recognizable. All analyses were performed using StatView (Version 5.0) or IBM SPSS® Statistics (Version 22.0) software. Significance was assumed at *P* < 0.05.

### Materials

1H‐[1,2,4]oxadiazolo‐[4,3‐a]quinoxalin‐1‐one (ODQ) was obtained from Enzo Life Sciences (Lausen, Switzerland) purchased through Eubio (Vienna, Austria). Apamin, charybdotoxin and iberiotoxin were purchased from Alomone Labs (Jerusalem, Israel). Collagenase (Worthington CLS 2) was from Worthington Biochemical Corporation (New York, USA). All other chemicals, including 5‐HMF, were of standard grade and purchased from Sigma‐Aldrich (Vienna, Austria).

### Nomenclature of targets and ligands

Key protein targets and ligands in this article are hyperlinked to corresponding entries in http://www.guidetopharmacology.org, the common portal for data from the IUPHAR/BPS Guide to PHARMACOLOGY (Southan *et al.,* 2016), and are permanently archived in the Concise Guide to PHARMACOLOGY 2015/16 (Alexander *et al.,* 2015a,b).

## Results

### Effect of 5‐HMF on vascular tone

**Figure 1:**
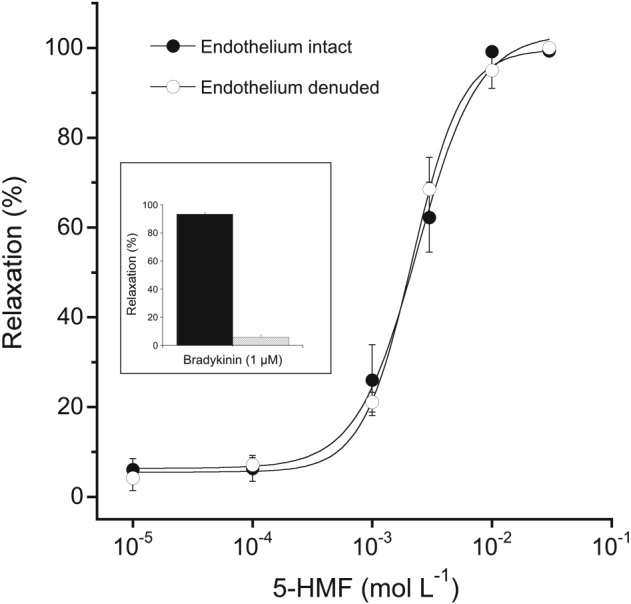
Concentration–response curves of 5‐HMF‐induced vasodilation in porcine coronary arteries with either intact or denuded endothelium. Rings were precontracted with the thromboxane mimetic U‐46619 (50 nM) and cumulative concentration–response curves to 5‐HMF were established. Effective denudation of the endothelium was confirmed by a lack of relaxation to bradykinin (1 μM; inset). Concentration–response curves established with different ring segments from a single heart were averaged and counted as an individual experiment. Data are mean values ± SEM of six hearts.

Myography experiments were performed to test for possible effects of 5‐HMF on vascular tone. As shown in Figure 1, 5‐HMF caused relaxation of U‐46619 precontracted porcine coronary arteries in a concentration‐dependent manner with an EC_50_ value of 2.6 ± 0.4 mM. Removal of the endothelium, a procedure that led to 80% loss of vasodilation to the endothelium‐dependent receptor agonist bradykinin (inset to Figure 1), did not affect 5‐HMF‐induced relaxation, excluding an involvement of the vascular endothelium in the observed vasodilation (for representative traces, see Supporting Information Figure S1).

To further clarify the mechanism of 5‐HMF‐induced relaxation, several pathways that have been shown to mediate vascular tone were blocked with inhibitors of enzymes and ion channels. The effect of the drug persisted in the presence of ODQ (100 μM), indomethacin (10 μM) and baicalein (10 μM), in order to exclude the involvement of soluble guanylate cyclase, cyclooxygenase and lipoxygenase respectively. Blocking small, intermediate or large Ca^2+^‐activated K^+^ channels with apamin (100 nM), charybdotoxin (100 nM) or iberiotoxin (100 nM), respectively, did not alter vasodilation to 5‐HMF. Furthermore, inhibition of sarcolemmal ATP‐sensitive K^+^ channels with glibenclamide (10 μM) or Na^+^/K^+^‐ATPase with ouabain (100 μM) had no effect (data not shown).

### Effect of 5‐HMF on normoxic heart function

**Figure 2:**
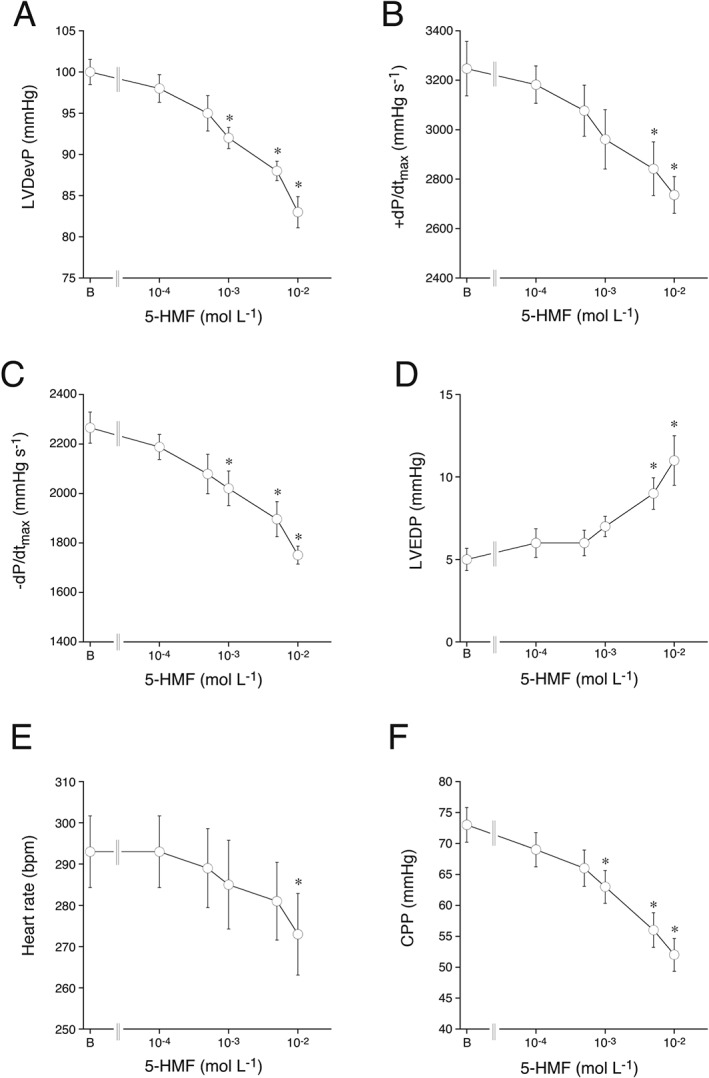
Cardiac effects of 5‐HMF on LVDevP (A), +dP/dt_max_ (B), −dP/dt_max_ (C), LVEDP (D), heart rate (E) and CPP (F) of isolated Langendorff‐perfused rat hearts. Data are mean values ± SEM of six hearts. *P < 0.05, significantly different from basal function (indicated as B); Student's *t*‐test.

To get more detailed information on the cardiovascular effects of 5‐HMF, the substance was applied to rat isolated perfused hearts. The effects of 5‐HMF on basal heart function are shown in Figure 2. At concentrations ≥1 mM, 5‐HMF exhibited concentration‐dependent negative inotropy (∆ decrease with 10 mM 5‐HMF for LVDevP and +dP/dt_max_: −17 ± 3 mmHg and −511 ± 62 mmHg·s^−1^, respectively) and lusitropy (∆ decrease for −dP/dt_max_: −515 ± 71 mmHg·s^−1^; Figure 2A–C; Supporting Information Figure S2). It also significantly increased LVEDP (∆ increase with 10 mM 5‐HMF: +6 ± 1 mmHg; Figure 2D; Supporting Information Figure S2). Finally, higher concentrations of 5‐HMF caused a significant reduction of heart rate (∆ decrease in heart rate with 10 mM 5‐HMF: −20 ± 2 bpm; Figure 2E; Supporting Information Figure S2). As already observed with porcine coronary arteries, 5‐HMF relaxed rat coronary microvessels, decreasing the CPP of hearts perfused at constant flow (∆ with10 mM 5‐HMF: −10 ± 2 mmHg; Figure 2F).

### Effect of 5‐HMF on cardiac function in I/R

**Figure 3:**
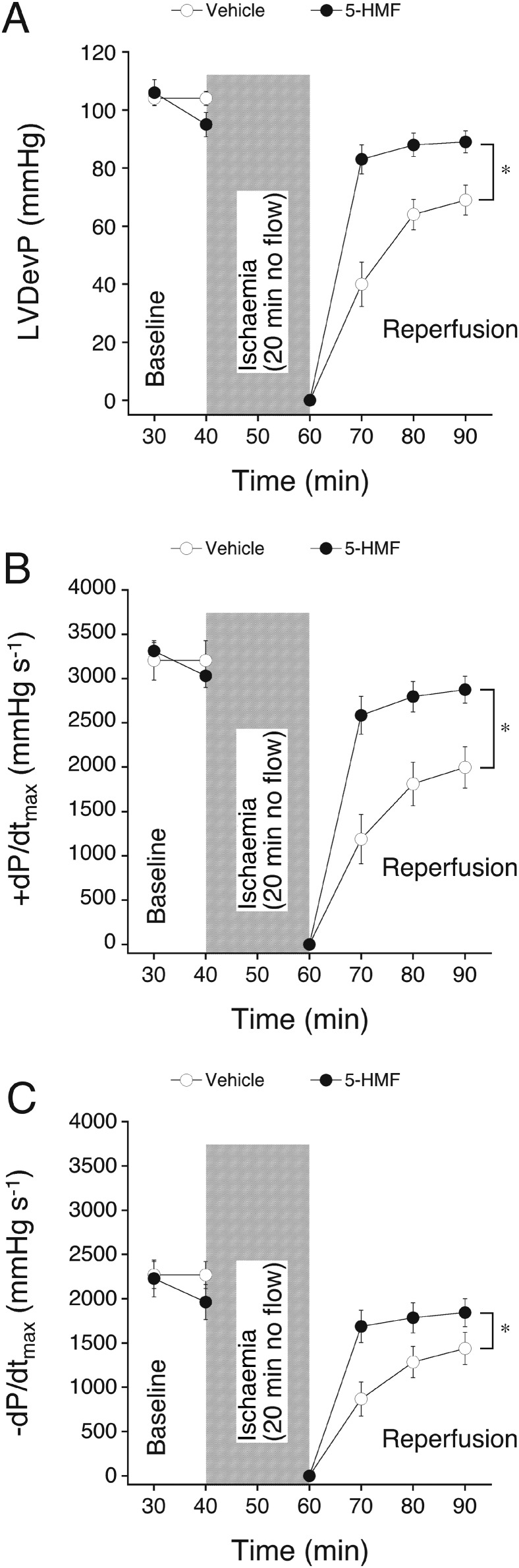
Effect of 5‐HMF on contractile function of isolated hearts subjected to 20 min no flow ischaemia, followed by reperfusion. 5‐HMF (5 mM) was added to the perfusion medium 10 min prior to induction of ischaemia. Effects on LVDevP (A), +dP/dt_max_ (B) and −dP/dt_max_ (C) were compared to vehicle perfused hearts. Data are mean values ± SEM of six hearts. *P < 0.05, significantly different from vehicle; ANOVA.

The results from the normoxic heart perfusion experiments led us to study the effect of 5‐HMF on hearts subjected to I/R. Generally, no‐flow ischaemia resulted in cessation of left ventricular contraction within ~5 min, accompanied by an increase in LVEDP. After 30 min of reperfusion LVDevP, +dP/dt_max_ and −dP/dt_max_ remained depressed to 65% in control hearts, while hearts that had been treated with 5‐HMF (5 mM) almost completely recovered to pre‐ischaemic levels. Notably, despite the slight negative inotropic and lusitropic action of 5‐HMF under normoxia, 5‐HMF treatment also improved recovery of inotropic and lusitropic parameters in absolute terms compared to untreated hearts (Figure 3; representative recordings are presented in Supporting Information (Figure S3).

**Figure 4:**
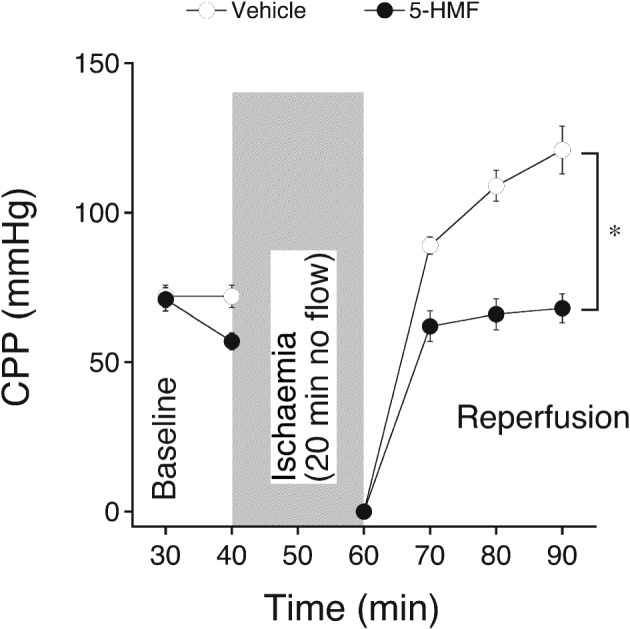
Effect of 5‐HMF on reperfusion coronary arterial function of isolated hearts subjected to 20 min no flow ischaemia, followed by reperfusion. 5‐HMF (5 mM) was added to the perfusion medium 10 min prior to induction of ischaemia. Effects on CPP were compared to vehicle perfused hearts. Data are mean values ± SEM of six hearts. *P < 0.05, significantly different from vehicle; ANOVA.

**Figure 5:**
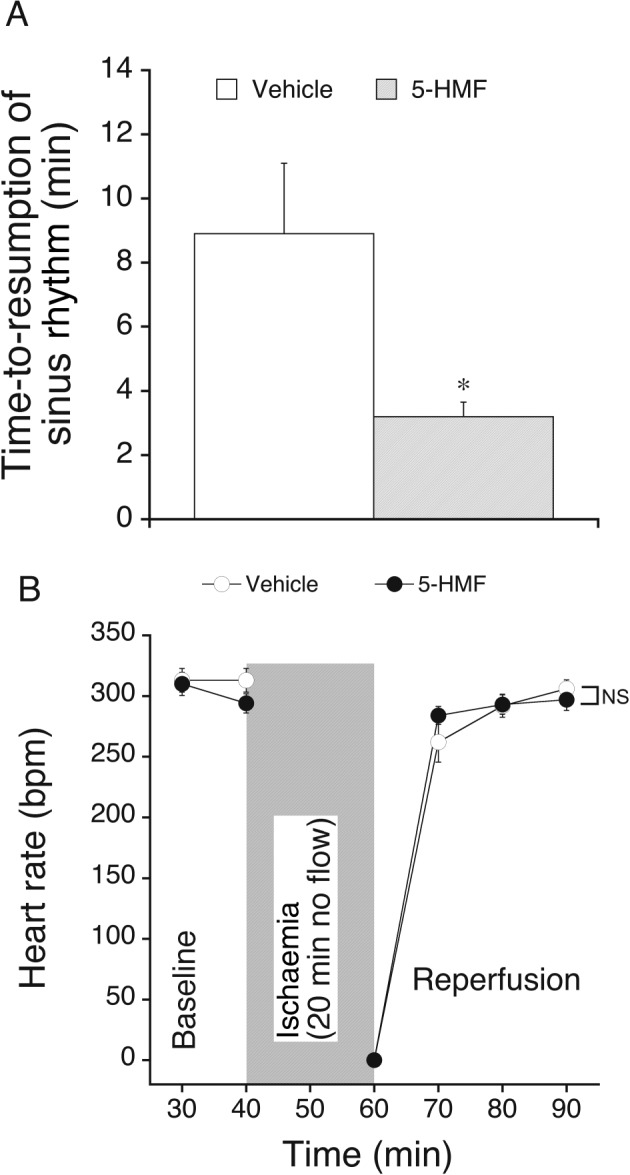
Effect of 5‐HMF on time‐to‐resumption of a stable sinus rhythm after initiation of reperfusion (A) and heart rate (B) of isolated hearts subjected to 20 min no flow ischaemia, followed by reperfusion. 5‐HMF (5 mM, filled circles) was added to the perfusion medium 10 min prior to induction of ischaemia. Effects were compared to vehicle (open circles) perfused hearts. *P < 0.05 versus vehicle (*t*‐test). Data are mean values ± SEM of six hearts. NS, non significant.

Perfusion with 5‐HMF did not alter the increase in end‐diastolic pressure during ischaemia and reperfusion compared to vehicle‐perfused hearts. Peak contracture and time‐to‐onset of ischaemic contracture were 45 ± 6% and 9.2 ± 0.8 min in vehicle and 48 ± 6% and 9.3 ± 0.9 min in 5‐HMF‐perfused hearts respectively (see Methods for calculation of these parameters). The increase in CPP after 30 min reperfusion in control hearts was abolished in hearts treated with 5‐HMF, indicating that the vasorelaxant effect of 5‐HMF persisted after ischaemia and probably contributed to the observed cardioprotection (Figure 4). As expected, reperfusion of hearts resulted in the occurrence of massive arrhythmias in the early reperfusion phase that ceased during reperfusion (see Supporting Information Figure S3). The time to resumption of a stable sinus rhythm in the two experimental groups is shown in Figure 5A. Importantly, hearts treated with 5‐HMF re‐adopted a stable sinus rhythm earlier than control hearts, although heart rate during reperfusion was not different between experimental groups (Figure 5B). Note that the heart rate in normoxic‐perfused hearts was neither significantly affected by 5 mM 5‐HMF (compare Figure 2E).

### Effect of 5‐HMF on I_Ca,L_

**Figure 6:**
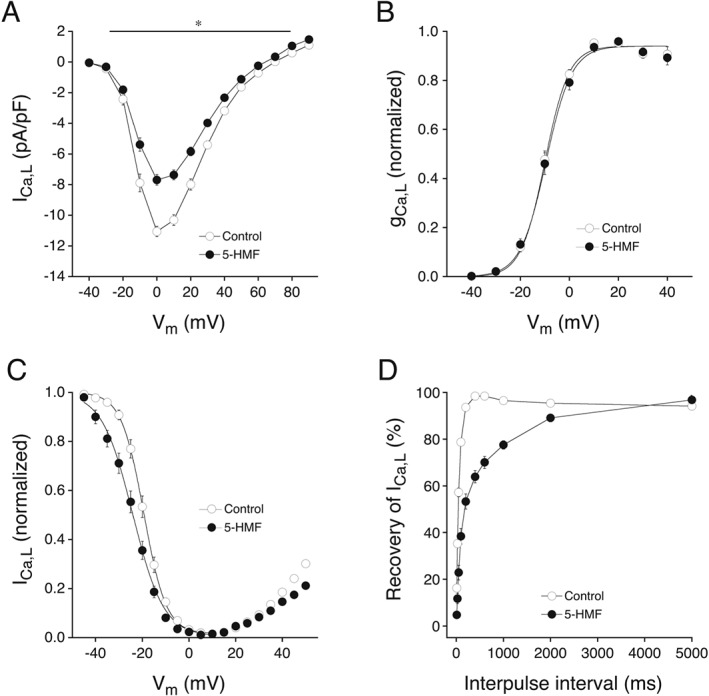
Effect of 5‐HMF on current density (A), steady‐state activation (B), steady‐state inactivation (C) and % recovery from inactivation (D) of I_Ca,L_ in GPV cardiomyocytes before (open circles) and after 1 mM 5‐HMF perfusion (5 min). I_Ca,L_ was elicited by a train of voltage pulses from −40 to +90 mV (10 mV interval, 400 ms) preceded by a pre‐pulse from −80 to −40 mV (50 ms). To determine steady‐state inactivation, I_CaL_ was activated with test pulses to +10 mV (400 ms) preceded by conditioning prepulses from −45 to +50 mV (5 mV increment, 400 ms). The amplitude of I_Ca,L_ during the test pulse was normalized to the highest value and plotted as a function of prepulse potential. The steady‐state inactivation curve was fitted within a V_m_ range from −45 to +10 mV. The % recovery from inactivation of I_Ca,L_ was examined using two depolarizing pulses to +10 mV with varying inter‐pulse intervals (10–5000 ms) applied from a holding potential of −40 mV every 8 s. Measurements on multiple cells derived from a single animal were averaged and counted as an individual experiment. Values are expressed as mean ± SEM of *n* experiments. *n* = 9 (A), 8 (B), 10 (C) and 7 (D). *P < 0.05, significantly different from control; paired *t*‐test.

The functional experiments suggest that 5‐HMF exhibits typical characteristics of an inhibitor of I_Ca,L_. Patch‐clamp experiments with GPV myocytes were performed to confirm this assumption. Original I_Ca,L_ traces in the absence or presence of 5‐HMF (1 mM) indicated a decrease of I_Ca,L_ by 5‐HMF (compare Supporting Information Figure S4A). Analysis of mean current density to voltage relationships of I_Ca,L_ (Figure 6A) demonstrated a significant reduction of I_Ca,L_ density at most tested membrane potentials. For example, peak current density (at 0 mV) decreased from −11.1 ± 0.3 to −7.7 ± 0.3 pA/pF by 5‐HMF. Time‐matched experiments in the absence of 5‐HMF produced a negligible I_Ca,L_ rundown (data not shown). Biexponential time course of current inactivation was unaffected except at a membrane potential of −10 mV where 5‐HMF significantly increased the fast inactivation time constant (Supporting Information Figures S4B and S5). Steady‐state I_Ca,L_ activation (Figure 6B) and inactivation (Figure 6C) curves were derived after 5‐HMF application. While I_Ca,L_ steady‐state activation was not altered (V_1/2_ = −9.9 ± 0.9 mV, k = 4.9 ± 0.3 mV in control), 5‐HMF significantly shifted the I_Ca,L_ steady‐state inactivation curve to more negative membrane potentials and reduced the slope (V_1/2_ = −20.1 ± 1.2 mV, k = 4.5 ± 0.1 mV and V_1/2_ = −25.3 ± 1.6 mV, k = 5.9 ± 0.3 mV for control and 5‐HMF, respectively). Moreover, application of 5‐HMF resulted in a slower recovery of I_Ca,L_ from inactivation (Figure 6D). Half‐recovery time of I_Ca,L_ was significantly prolonged from 43.7 ± 1.8 to 168.5 ± 41.5 ms after addition of 5‐HMF.

**Figure 7:**
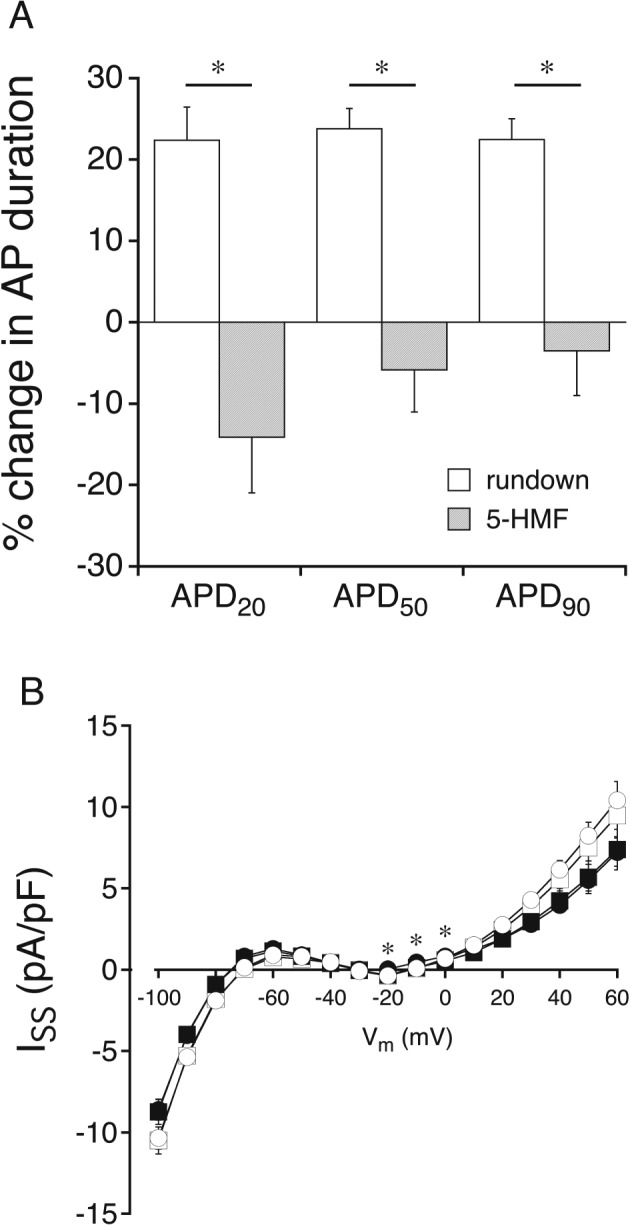
Effect of 5‐HMF on APD (A) and whole‐cell I_ss_ (B) in GPV cardiomyocytes. (A) AP recordings were made before and after 5‐HMF (1 mM) perfusion or rundown, and the difference in duration was calculated in %. (B) I_ss_ was elicited by slow (20 s) voltage ramps from −100 to +60 mV and measured before (open symbols) and after 5‐HMF perfusion or time‐matched rundown. Measurements on multiple cells derived from a single animal were averaged and counted as an individual experiment. Values are expressed as mean ± SEM of seven experiments. *P < 0.05, significantly different from time‐matched rundown; ANCOVA.

As I_Ca,L_ is essential for the maintenance of the AP plateau, we studied the effect of 5‐HMF on action potential duration (APD). Representative APs (Supporting Information Figure S6A) clearly demonstrate an overall decrease in APD in the presence of 5‐HMF. Compared to time‐matched rundown, 5‐HMF significantly reduced APD from 199.0 ± 21.9, 307.9 ± 26.6 and 357.3 ± 24.4 ms to 129.8 ± 23.3, 230.1 ± 26.9 and 272.4 ± 28.4 ms at 20, 50 and 90% of repolarization respectively. The percentage change of APD is shown Figure 7A. Resting membrane potential hyperpolarized by ~4 mV in time‐matched control experiments as well as after addition of 5‐HMF. AP upstroke velocity was unaffected by 5‐HMF indicating no influence on sodium current. To elucidate whether I_K_ are affected by 5‐HMF and thereby also contribute to the observed AP shortening, slow voltage ramps were performed. Supporting Information Figure S6B shows original I_ss_ in the absence and presence of 5‐HMF. A decrease in outward current density at positive membrane potentials and a shift of the reversal potential to more negative membrane potentials (reflecting the observed hyperpolarization of resting membrane potential) was not different between time‐matched rundown and 5‐HMF (Figure 7B). Therefore, it is reasonable to assume that neither the delayed rectifier I_K_ nor the inward rectifier I_K_ is affected by 5‐HMF. The significant difference at the potential range from −20 to 0 mV reflects the I_Ca,L_ reduction by 5‐HMF.

## Discussion

There is clear and convincing experimental evidence that oxidative injury contributes to the pathogenesis and progression of many cardiovascular complications, including ischaemic heart disease, hypertension and cardiac hypertrophy. Therefore, antioxidant supplementation in addition to established therapies with, for example, ß‐blockers, Ca^2+^ channel blockers or organic nitrates, is recommended for the treatment of cardiac diseases. However, attempts to translate our understanding of these molecular and cellular mechanisms into clinical practice have failed so far. A vast number of clinical trials report no benefit of antioxidant supplementation in the genesis or progression of cardiovascular diseases (see Steinhubl, 2008; Leopold, 2015). Various reasons might account for these failures, but taken together, the negative results clearly indicate that the use of antioxidant drugs in clinical applications requires precise knowledge of their physiological actions and underlying molecular mechanisms to understand their beneficial and avoid potential adverse effects. Interestingly, the best outcome from clinical trials was achieved by intervention with the Mediterranean diet that was attributed (in part) to a reduction in oxidative stress (Fito *et al.,* 2007; Estruch *et al.,* 2013), renewing the interest in studying dietary antioxidants. Among numerous other drugs, 5‐HMF, a natural ingredient of plants, foods and beverages, has recently been considered as an efficient antioxidant (Li *et al.,* 2009). Continuing *in vitro* studies, reporting antioxidative, antiproliferative and antiinflammatory properties (Kim *et al.,* 2011; Zhao *et al.,* 2013), emphasized the antioxidative potential of 5‐HMF but did not consider additional mechanisms. In view of the therapeutic potential of 5‐HMF as a dietary supplement (Matzi *et al.,* 2007; Gatterer *et al.,* 2013; Mariacher *et al.,* 2014) and as an anti‐sickling drug (Abdulmalik *et al.,* 2005; Safo and Kato, 2014), we examined the cardiovascular effects of 5‐HMF.

### Cardiovascular effects of 5‐HMF are (primarily) mediated by inhibition of I_Ca,L_

In the low millimolar range, 5‐HMF relaxed coronary arteries in an endothelium‐independent manner that could not be explained by scavenging of superoxide or a related antioxidant action. Since vasodilation to 5‐HMF was resistant to inhibition of enzymes typically involved in smooth muscle relaxation, that is, soluble guanylate cyclase, cyclooxygenase and lipoxygenase, we considered that the drug may directly act on ion channels. Pharmacological interference with Ca^2+^‐activated K^+^ channels, ATP‐sensitive K^+^ channels and Na^+^/K^+^‐ATPase did not affect 5‐HMF‐induced relaxation, suggesting a selective effect of 5‐HMF on L‐type Ca^2+^ channels that was confirmed in electrophysiological experiments. Importantly, patch clamp experiments in cardiac myocytes revealed that 5‐HMF significantly reduced I_Ca,L_, shifted the steady‐state inactivation curve to more negative membrane potentials and slowed down recovery of I_Ca,L_ from inactivation, thereby functionally reducing I_Ca,L_. Interestingly, biexponential time course of current inactivation was unaffected except τ_f_ at a membrane potential of −10 mV being increased by 5‐HMF. It is widely assumed that I_Ca,L_ inactivation follows a biexponential time course with a fast initial decay representing the Ca^2+^‐dependent inactivation (τ_f_) and a slower phase reflecting the voltage‐dependent inactivation (τ_s_) (Benitah *et al.,* 2010; Grandi *et al.,* 2010). Ca^2+^‐dependent inactivation is mainly due to Ca^2+^ released from the sarcoplasmic reticulum *via* ryanodine receptors and to a lesser extent by Ca^2+^ entry through the L‐type channels (Adachi‐Akahane *et al.,* 1996; Sham, 1997). If Ca^2+^ release from the sarcoplasmic reticulum is unchanged by 5‐HMF, a moderate reduction of Ca^2+^ entry *via* L‐type channels would only slightly alter the time course of current decay and is therefore possibly not measurable in our experimental design. Moreover, it is known that some Ca^2+^ channel blockers bind to the channel in the inactivated state and hence do not affect the time course of calcium current decay (Nawrath and Wegener, 1997; Ogura *et al.,* 1999), which could be another reasonable explanation for our results. Further studies are needed to shed light on the mode of 5‐HMF action.

The observed shortening of APD is most likely due to the I_Ca,L_ impairment since delayed rectifier I_K_ and inward rectifier I_K_ appear to be unaffected. However, it cannot be excluded that 5‐HMF also affects other ion currents. These observations reveal a novel mechanism of action of 5‐HMF that adds to the well‐known antioxidant properties of this compound.

In rat isolated normoxic‐perfused hearts, 5‐HMF exerted typical characteristics of a Ca^2+^ channel blocker (i.e. negativ inotropy, lusitropy, chronotropy and coronary vasodilation) (Eisenberg *et al.,* 2004). Voltage‐gated Ca^2+^ channels are essentially involved in excitation‐contraction coupling of cardiac and smooth muscle. Inhibition of Ca^2+^ entry results in reduced myocardial contractility and vasodilation. Although not studied in detail, the observed reduction of heart rate is most likely caused by an inhibitory action of 5‐HMF on L‐type Ca^2+^ channels in the sinoatrial node, where I_Ca,L_ essentially contributes to diastolic depolarization underlying the spontaneous generation of APs (Katz, 1986). Notably, effects were clearly less pronounced in cardiac myocytes (e.g. 20% reduction of inotropy with 10 mM 5‐HMF) as compared to smooth muscle cells (100% vasodilation with 10 mM 5‐HMF), implying that the overall haemodynamic profile of 5‐HMF might be dominated by a reduction of blood pressure, with the negative inotropic and chronotropic actions possibly being compensated by the baroreflex, *in vivo*. However, the negative inotropic potential has to be considered especially when there may be pre‐existing pathologies with impaired sympathetic tone and cardiovascular reflexes. This also applies to ventricular diastolic function. In our experimental system, 5‐HMF decreased the velocity of diastolic relaxation and increased LVEDP. A slight acute increase in LVEDP after addition of 5‐HMF is difficult to reconcile with an inhibitory action on Ca^2+^ channels, but has been observed by others using established Ca^2+^ channel blockers *in vitro* (Amende *et al.,* 1992; Schmidlin *et al.,* 1992; Hara *et al.,* 2001) and *in vivo* (Kass *et al.,* 1993; Nishimura *et al.,* 1993; Cockrill *et al.,* 2001). It should be noted that these acute effects have to be distinguished from the often observed protective effects after long‐term treatment with Ca^2+^ channel blockers on diastolic dysfunction, attributed to indirect haemodynamic improvements (Aziz *et al.,* 2013). Moreover, it has been suggested that Ca^2+^ channel blockers applied at relatively high concentrations interfere with the Ca^2+^ uptake and release activity of the sarcoplasmic reticulum in cardiac and skeletal myocytes (Wang *et al.,* 1984). As 5‐HMF is cell‐permeable and has been shown to reduce intracellular oxidative stress (Li *et al.,* 2009) and to bind to intracellular HbS (Abdulmalik *et al.,* 2005), it is also conceivable that the acute increase in LVEDP might be caused by intracellular actions of 5‐HMF, unrelated to inhibition of Ca^2+^ channels.

### Cardioprotective effects in I/R suggest 5‐HMF as an addition to cardioplegic solutions

Because a burst of superoxide anions and intracellular Ca^2+^ overload is associated with cardiac I/R injury (Murphy and Steenbergen, 2008), the combined action of 5‐HMF as intracellular antioxidant and Ca^2+^ channel blocker renders this compound an ideal candidate for myocardial protection against I/R injury. This is reinforced by our observation of considerable cardioprotective effects of 5‐HMF in isolated hearts subjected to I/R. Importantly, the observed protection not only comprised increased cardiac contractility and enhanced coronary blood flow but also early resumption of a stable sinus rhythm after initiation of reperfusion. In view of the occurrence of life‐threatening arrhythmias as a common complication after heart transplantation (Hamon *et al.,* 2014) and preliminary data obtained previously with a refined 5‐HMF‐containing formulation (Schwarz *et al.,* 2012), the use of 5‐HMF as an addition to cardioplegic solutions is worth considering. Work is ongoing in our laboratories to confirm the beneficial effects of the drug in a clinical setting of cardiac I/R.

Ca^2+^ channel blockers were extensively tested as additives to cardioplegic solutions to reduce myocardial injury, and divergent results were obtained,depending on experimental study parameters (Murphy and Wechsler, 1987). Although clinical studies have failed to demonstrate convincing benefits of Ca^2+^ channel blockers in cardioplegic solutions so far (Christakis *et al.,* 1986; Flameng *et al.,* 1986), these drugs have remained attractive candidates as constituents of new cardioplegic formulations despite adverse effects, like depressed systolic function and atrioventricular block (Fallouh *et al.,* 2009). A serious obstacle for the use of Ca^2+^ channel blockers as additives to cardioplegic solutions is their long plasma half‐life and high affinity binding to L‐type Ca^2+^ channels (Scholz, 1997), both resulting in long‐lasting negative inotropy after resuscitation. The use of 5‐HMF, which is less potent and rapidly metabolized, might be advantageous for this application.

### In vivo implications and limitations

In view of the observed low potency of 5‐HMF in the *ex vivo* experiments, conclusions regarding possible implications for the *in vivo* situation should be drawn with caution, as long as definitive pharmacokinetic data are not available. *In vitro,* inhibition of Ca^2+^ channels by 5‐HMF occurs in a similar (millimolar) concentration range as reported for the antisickling (Abdulmalik *et al.,* 2005) and antioxidant (Li *et al.,* 2009; Kim *et al.,* 2011; Zhao *et al.,* 2013; Zhang *et al.,* 2015) actions of the drug. As an additive to cardioplegic solutions, 5‐HMF can be applied at high concentrations as required, but bioavailability might be an issue for oral administration to patients. In animal studies, a single oral application of 5‐HMF resulted in millimolar peak plasma concentrations (Abdulmalik *et al.,* 2005), accompanied by prolonged survival time of transgenic sickle mice. A commercial formulation of 5‐HMF contains high concentrations of the compound (Sanopal®, daily dose 750 mg), suggesting high bioavailability and potential *in vivo* relevance of the effects that were observed *in vitro* with fairly high 5‐HMF concentrations. At least in the study by Abdulmalik *et al*. (2005), the *in vitro* antisickling effect that was observed at similarly high concentrations was successfully translated to an *in vivo* animal model. One of us (A.O.) has developed an analytical method for quantification of 5‐HMF in plasma (Donnarumma *et al.,* 2013), and the pharmacokinetics of the compound is currently being studied in clinical trials. In the I/R experiments, 5‐HMF was added before induction of ischaemia, which of course does not reflect the clinical situation of acute myocardial infarction. Much more potent therapeutic agents are available for this specific application, but a prophylactic application to high‐risk patients might be indicated. Finally, it should be noted that, as no measurements of intracellular Ca^2+^ levels were performed in our experiments, some of the observed effects might be unrelated to inhibition of Ca^2+^ channels and rather caused by changes of intracellular Ca^2+^ handling.

Taken together, our unexpected observation that 5‐HMF inhibits I_Ca,L_ extends our understanding of the actions of 5‐HMF and sheds new light on the observed beneficial effects of the drug in several pathologies, like acute hypobaric hypoxia (Li *et al.,* 2011), alcoholic liver oxidative injury (Li *et al.,* 2015) and neurodegenerative diseases (Liu *et al.,* 2014a; Lee *et al.,* 2015; Zhang *et al.,* 2015). The cardiovascular effects reported in this study broaden the range of potential applications of 5‐HMF and suggest that the drug may be beneficial as an additive to cardioplegic solutions.

## Author contributions

G.W., C.N.K. and S.S. performed experiments and analysed data. A.S., A.O., G.W. and B.M. designed and managed the study. B.P., M.A., E.M. and K.Z. interpreted and discussed data. G.W., A.S. and B.M. wrote the manuscript. All authors contributed to manuscript preparation.

## Conflict of interest

The authors declare no conflicts of interest.

## Declaration of transparency and scientific rigour

This Declaration acknowledges that this paper adheres to the principles for transparent reporting and scientific rigour of preclinical research recommended by funding agencies, publishers and other organisations engaged with supporting research.

## Supporting information


**Figure S1** Representative traces of 5‐HMF‐induced vasodilation in porcine coronary arteries with either intact (A) or denuded (B) endothelium. Rings were precontracted with the thromboxane mimetic U‐46619 (50 nM) and 5‐HMF was added at the indicated timepoints (arrows) to the bathing solution (Concentrations in mol L^−1^; cumulative dosing).
**Figure S2** Representative recording of the effects of 5‐HMF on left‐ventricular pressure (LVP) and heart rate (HR) of normoxic perfused rat hearts. After equilibration, increasing doses of 5‐HMF were added as boli (upstrokes in line diagram) to the perfusion medium to establish a concentration–response curve (maximal achieved concentrations are given in the plateaus of the line diagram).
**Figure S3** Representative left‐ventricular pressure (LVP) recordings of isolated hearts subjected to 20 min no flow ischaemia followed by 30 min reperfusion. Vehicle (A) or 5‐HMF (5 mM; B) was added to the perfusion medium 10 min prior to induction of ischaemia.
**Figure S4** I_Ca,L_ traces before and after 5‐HMF (1 mM) perfusion. (A) Original current traces in response to voltage pulses ranging from −30 mV to +40 mV are shown for control (left panel) and 5‐HMF (right panel). (B) Calcium currents at the indicated test potentials were normalized to their corresponding peak value to make the time course of current decay comparable between control (black line) and 5‐HMF (grey line) conditions.
**Figure S5** Effect of 5‐HMF on I_Ca,L_ inactivation time course. (A) Representative traces of I_Ca,L_ decay (black line) elicited by a test potential to 0 mV before (left panel) and after 5‐HMF (1 mM) perfusion (right panel). Current decay was well described by a biexponential model (red line, goodness of fit was assessed by the adjusted R‐Square value). Inset shows the voltage clamp protocol. (B) Biexponential time course of I_Ca,L_ inactivation with a fast (τ_f_) and a slow (τ_s_) time constant at indicated test potentials. Measurements on multiple cells derived from a single animal were averaged and counted as an individual experiment. Values are expressed as mean ± SEM of 9 experiments. *P < 0.05 versus control (paired *t*‐test).
**Figure S6** Original recordings of action potentials (A) and whole cell I_ss_ (B) before and after 5‐HMF perfusion in a single isolated cell.
