# Supplementary material for: Cardioprotective effects of 5‐hydroxymethylfurfural mediated by inhibition of L‐type Ca2+ currents
Source: Br J Pharmacol. 2017 Sep 9;174(20):3640–53. doi: 10.1111/bph.13967 (PMC5610158; doi:10.1111/bph.13967)
Supplement: Supplementary file 1 — Figure S1 Representative traces of 5‐HMF‐induced vasodilation in porcine coronary arteries with either intact (A) or denuded (B) endothelium. Rings were precontracted with the thromboxane mimetic U‐46619 (50 nM) and 5‐HMF was added at the indicated timepoints (arrows) to the bathing solution (Concentrations in mol L−1; cumulative dosing). Figure S2 Representative recording of the effects of 5‐HMF on left‐ventricular pressure (LVP) and heart rate (HR) of normoxic perfused rat hearts. After equilibration, increasing doses of 5‐HMF were added as boli (upstrokes in line diagram) to the perfusion medium to establish a concentration–response curve (maximal achieved concentrations are given in the plateaus of the line diagram). Figure S3 Representative left‐ventricular pressure (LVP) recordings of isolated hearts subjected to 20 min no flow ischaemia followed by 30 min reperfusion. Vehicle (A) or 5‐HMF (5 mM; B) was added to the perfusion medium 10 min prior to induction of ischaemia. Figure S4 ICa,L traces before and after 5‐HMF (1 mM) perfusion. (A) Original current traces in response to voltage pulses ranging from −30 mV to +40 mV are shown for control (left panel) and 5‐HMF (right panel). (B) Calcium currents at the indicated test potentials were normalized to their corresponding peak value to make the time course of current decay comparable between control (black line) and 5‐HMF (grey line) conditions. Figure S5 Effect of 5‐HMF on ICa,L inactivation time course. (A) Representative traces of ICa,L decay (black line) elicited by a test potential to 0 mV before (left panel) and after 5‐HMF (1 mM) perfusion (right panel). Current decay was well described by a biexponential model (red line, goodness of fit was assessed by the adjusted R‐Square value). Inset shows the voltage clamp protocol. (B) Biexponential time course of ICa,L inactivation with a fast (τf) and a slow (τs) time constant at indicated test potentials. Measurements on multiple cells derived from a single ani [file BPH-174-3640-s001.pdf]

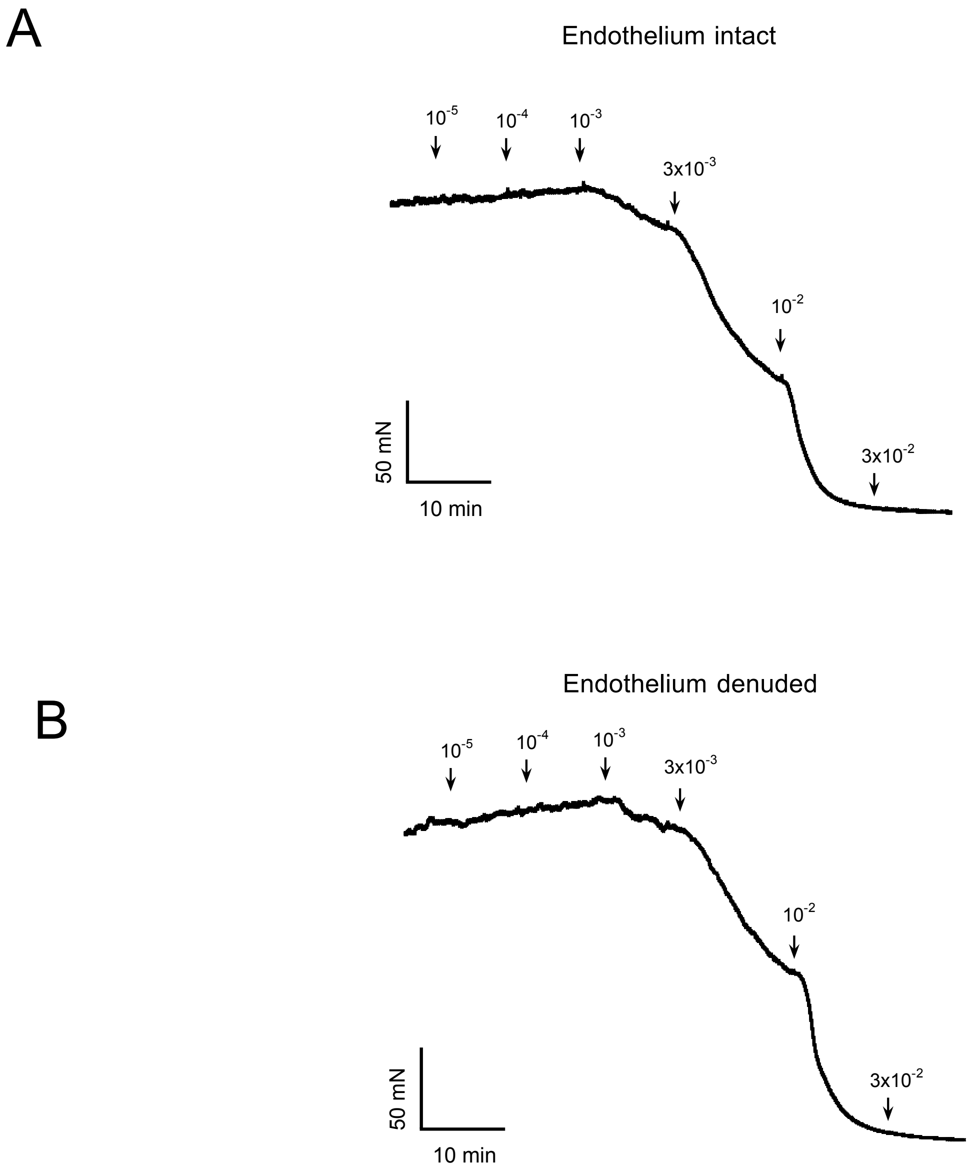

Supplementary Figure 1 Representative traces of 5-HMF-induced vasodilation in porcine coronary arteries with either intact (A) or denuded (B) endothelium. Rings were precontracted with the thromboxane mimetic U-46619 (50 nM) and 5-HMF was added at the indicated timepoints (arrows) to the bathing solution (Concentrations in mol L^{-1}; cumulative dosing).

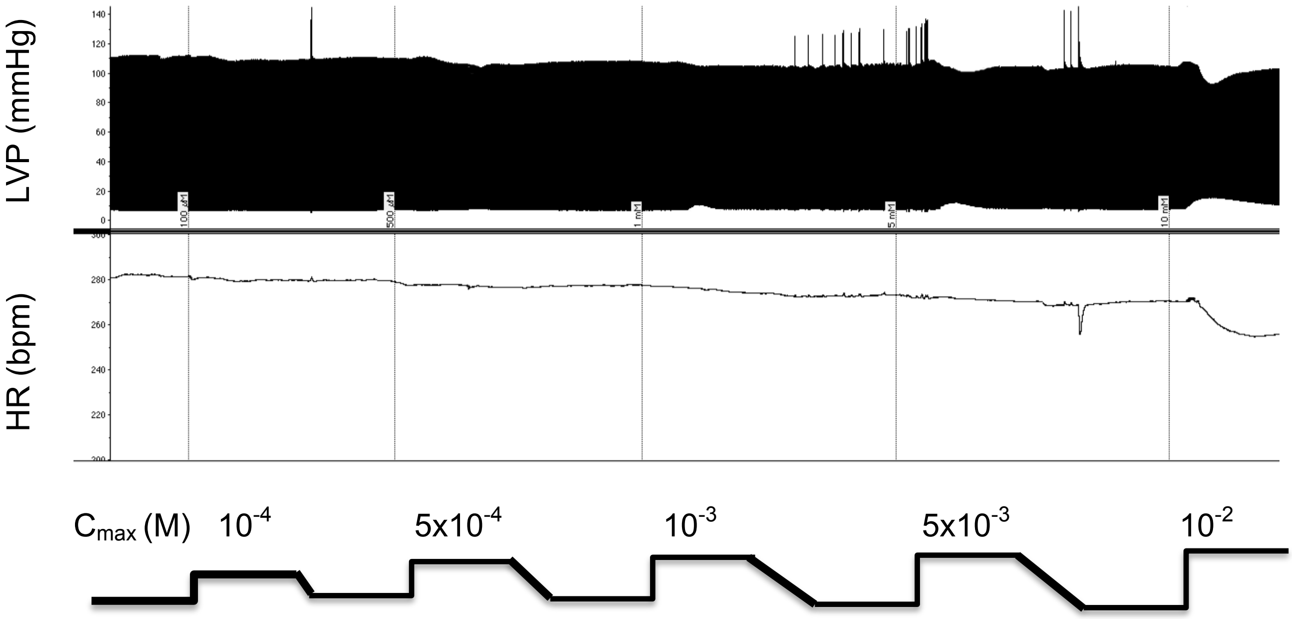

Supplementary Figure 2 Representative recording of the effects of 5-HMF on left-ventricular pressure (LVP) and heart rate (HR) of normoxic perfused rat hearts. After equilibration increasing doses of 5-HMF were added as boli (upstrokes in line diagram) to the perfusion medium to establish a concentration-response curve (maximal achieved concentrations are given in the plateaus of the line diagram).

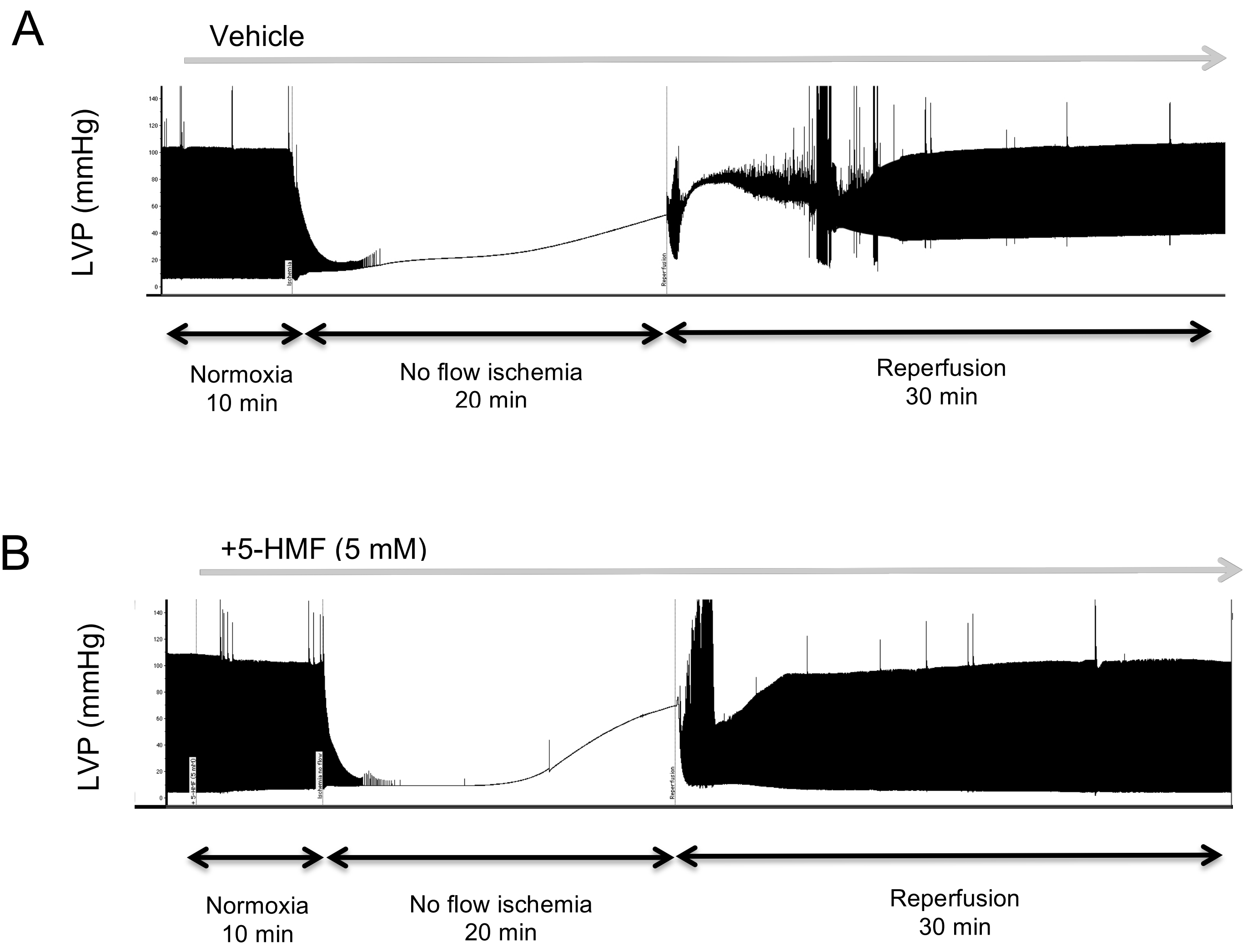

Supplementary Figure 3 Representative left-ventricular pressure (LVP) recordings of isolated hearts subjected to 20 min no flow ischemia followed by 30 min reperfusion. Vehicle (A) or 5-HMF (5 mM; B) was added to the perfusion medium 10 min prior to induction of ischemia.

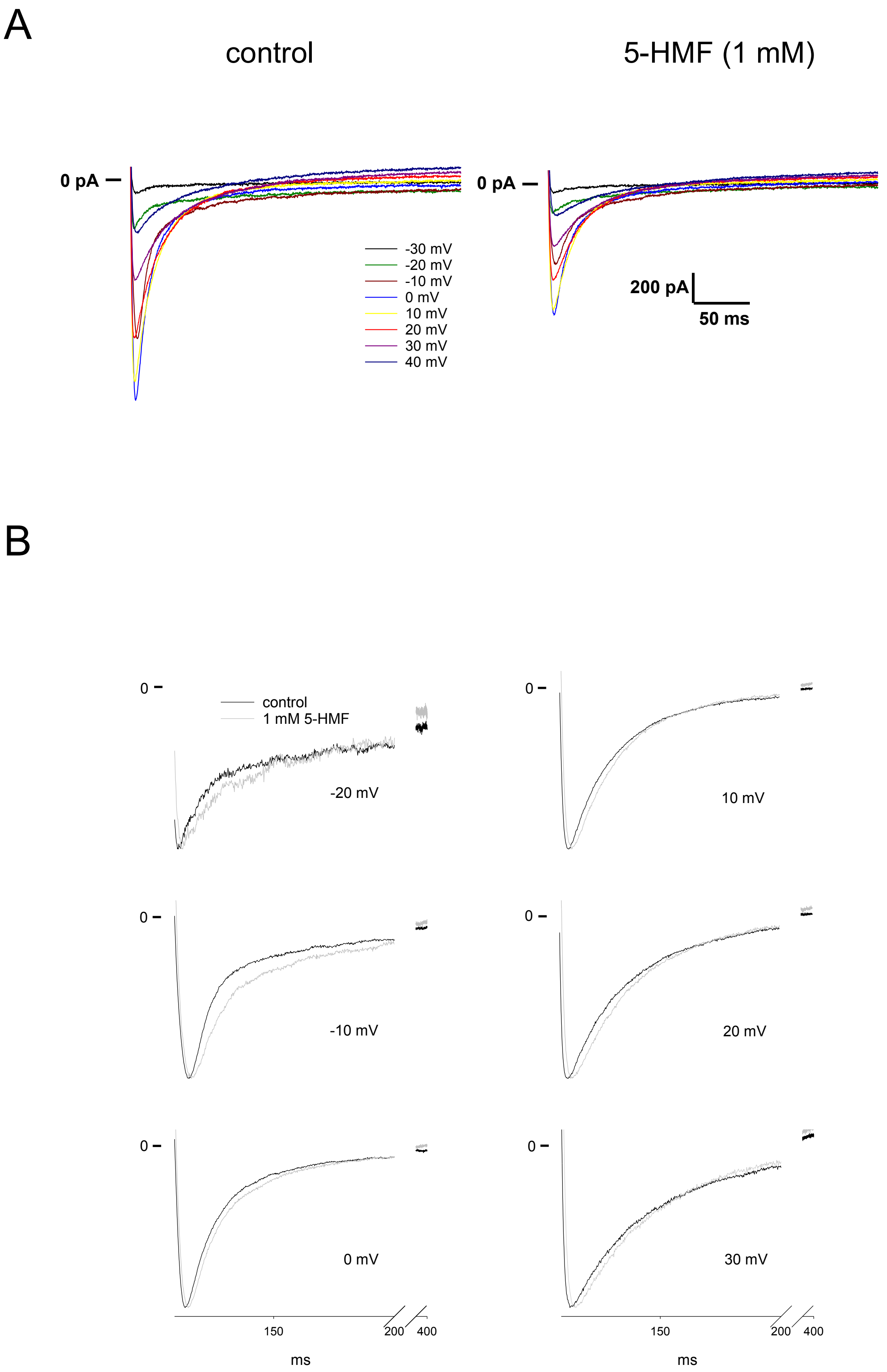
A
control
5-HMF (1 mM)
0 pA
0 pA
-30 mV
-20 mV
-10 mV
0 mV
10 mV
20 mV
30 mV
40 mV
200 pA
50 ms
B
control
1 mM 5-HMF
-20 mV
10 mV
-10 mV
20 mV
0 mV
30 mV
0
150
200
400
ms

Supplementary Figure 4 $I_{Ca,L}$ traces before and after 5-HMF (1mM) perfusion. (A) Original current traces in response to voltage pulses ranging from -30 mV to +40 mV are shown for control (left panel) and 5-HMF (right panel). (B) Calcium currents at the indicated test potentials were normalized to their corresponding peak value to make the time course of current decay comparable between control (black line) and 5-HMF (grey line) conditions.

A

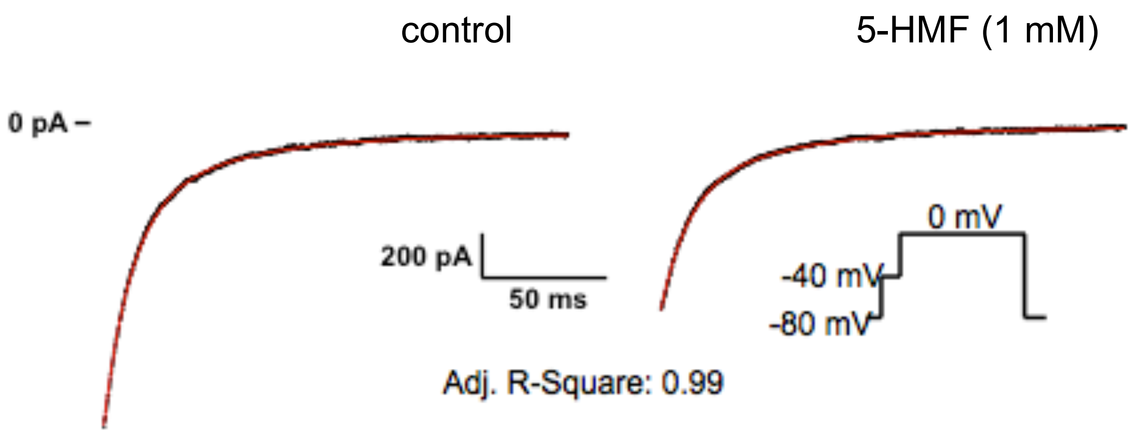

B

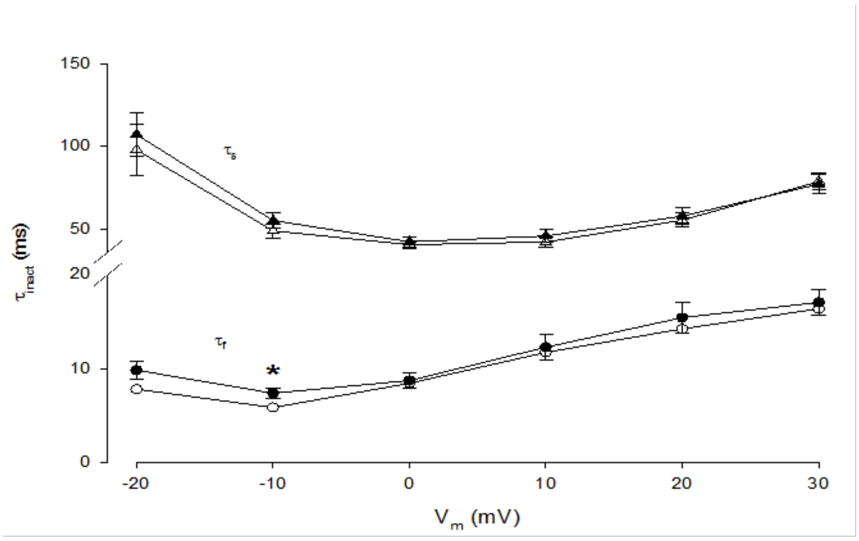

Supplementary Figure 5 Effect of 5-HMF on $I_{Ca,L}$ inactivation time course. (A) Representative traces of $I_{Ca,L}$ decay (black line) elicited by a test potential to 0 mV before (left panel) and after 5-HMF (1 mM) perfusion (right panel). Current decay was well described by a biexponential model (red line, goodness of fit was assessed by the adjusted R-Square value). Inset shows the voltage clamp protocol. (B) Biexponential time course of $I_{Ca,L}$ inactivation with a fast (τ_f) and a slow (τ_s) time constant at indicated test potentials. Measurements on multiple cells derived from a single animal were averaged and counted as an individual experiment. Values are expressed as mean ± SEM of 9 experiments. *$P<0.05$ versus control (paired *t*-test).

A

B

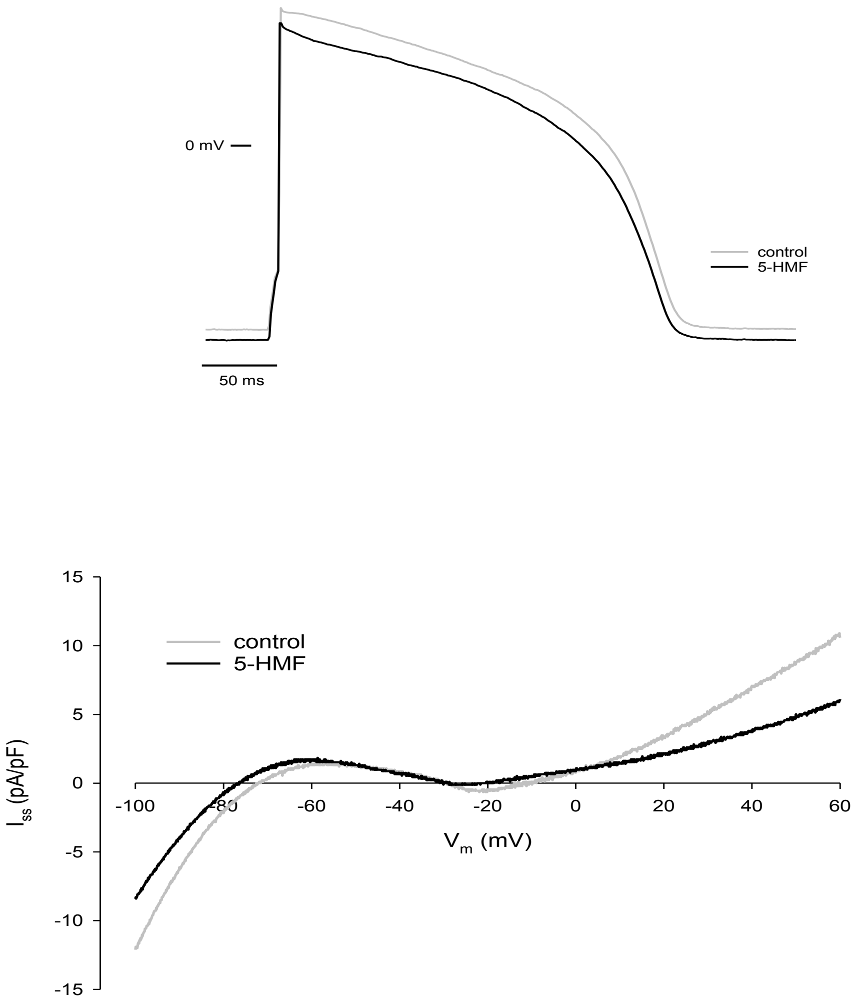

Supplementary Figure 6 Original recordings of action potentials (A) and whole cell I_{SS} (B) before and after 5-HMF perfusion in a single isolated cell.
